# Rapid Dissemination of SIV Follows Multisite Entry after Rectal Inoculation

**DOI:** 10.1371/journal.pone.0019493

**Published:** 2011-05-09

**Authors:** Patricia Ribeiro dos Santos, Magali Rancez, Jean-Luc Prétet, Alice Michel-Salzat, Valérie Messent, Anna Bogdanova, Anne Couëdel-Courteille, Evelyne Souil, Rémi Cheynier, Cécile Butor

**Affiliations:** 1 Laboratoire de Transmission et Dissémination Virales, Université Paris Diderot, Sorbonne Paris Cité, Paris, France; 2 Plateforme de Morpho-Histologie, Institut Cochin, INSERM U1016, CNRS URA8104, Université Paris Descartes UMR-S1016, Paris, France; 3 Département d'Immunologie-Hématologie, Institut Cochin, INSERM U1016, CNRS URA8104, Université Paris Descartes UMR-S1016, Paris, France; Karolinska Institutet, Sweden

## Abstract

Receptive ano-rectal intercourse is a major cause of HIV infection in men having sex with men and in heterosexuals. Current knowledge of the mechanisms of entry and dissemination during HIV rectal transmission is scarce and does not allow the development of preventive strategies. We investigated the early steps of rectal infection in rhesus macaques inoculated with the pathogenic isolate SIVmac251 and necropsied four hours to nine days later. All macaques were positive for SIV. Control macaques inoculated with heat-inactivated virus were consistently negative for SIV. SIV DNA was detected in the rectum as early as four hours post infection by nested PCR for *gag* in many laser-microdissected samples of lymphoid aggregates and lamina propria but never in follicle-associated epithelium. Scarce SIV antigen positive cells were observed by immunohistofluorescence in the rectum, among intraepithelial and lamina propria cells as well as in clusters in lymphoid aggregates, four hours post infection and onwards. These cells were T cells and non-T cells that were not epithelial cells, CD68^+^ macrophages, DC-SIGN^+^ cells or fascin^+^ dendritic cells. DC-SIGN^+^ cells carried infectious virus. Detection of *Env* singly spliced mRNA in the mucosa by nested RT-PCR indicated ongoing viral replication. Strikingly, four hours post infection colic lymph nodes were also infected in all macaques as either SIV DNA or infectious virus was recovered. Rapid SIV entry and dissemination is consistent with trans-epithelial transport. Virions appear to cross the follicle-associated epithelium, and also the digestive epithelium. Viral replication could however be more efficient in lymphoid aggregates. The initial sequence of events differs from both vaginal and oral infections, which implies that prevention strategies for rectal transmission will have to be specific. Microbicides will need to protect both digestive and follicle-associated epithelia. Vaccines will need to induce immunity in lymph nodes as well as in the rectum.

## Introduction

Receptive rectal intercourse with an HIV^+^ individual carries a per act risk of transmission of 0.5% to 1.7% [1]–[3]. This is three to five-fold higher than receptive vaginal intercourse [3], [4]. In 2009 Men having Sex with Men (MSM) represented 59.5% of all new HIV infections in the USA [5], and 39.8% in Canada [6]. In males, MSM represent 78.4% of newly acquired infections in the USA [5], and 59.5% in Canada. MSM are the population group with the highest risk of acquiring HIV worldwide, including developing countries [7]. Rectal intercourse practiced in heterosexual relationships [2], [8]–[10] increases several fold the male to female transmission risk [11]–[13].

Additionally, an increase in severity of disease for MSM with respect to intravenous transmission has been noted in most but not all cohorts [14]–[31]. This phenomenon could be due to the prevalence of specific opportunistic pathogens in that population [31], such as HHV-8 [29], [30]. Alternatively, entry by the rectal route could modify the initial immune response and worsen the clinical course of HIV infection [14].

HIV can cross the rectal epithelium by several mechanisms: open lesion with direct access to blood leukocytes [32], [33], productive infection of epithelial cells [34]–[37], transcytosis by epithelial cells [38], [39] and/or M cells [40], [41], opening of tight junctions [42]–[44], and capture by intraepithelial dendritic cells (DC) [45]–[48]. Transport of the virus out of the mucosa to lymph nodes can be due to free virions in the lymph, infected cells or virions bound to DCs [49], the C-type lectin DC-SIGN being then a prime candidate for binding the virus [50]–[52].

For ethical reasons, in vivo data on viral entry and dissemination can only be obtained in animal models. Experimental infection of macaques with SIV currently represents the best animal model for HIV infection [53], [54]. Earlier work on rectal infection in rhesus and pigtail macaques showed that viral dissemination is rapid [55]–[59], proceeds in five stages with wide animal to animal variation in terms of kinetics [55], [56] and differs from intravenous entry [55], [56]. To our knowledge, no information was gained on the pathway followed by the virus for rectal entry. A good understanding of the mode of entry and of dissemination is important for the development of preventive strategies, whether chemical or vaccine based [44], [60]. We decided to address this question in the rhesus macaque (*Macaca mulatta*) following rectal infection with the pathogenic isolate SIVmac251. We used free virions as inoculum. Current data does suggest that during sexual HIV transmission the source virus more likely originates from free viral particles than from infected cells in seminal plasma [61], [62].

We show here that SIV is present in lymphoid aggregates as well as in the lamina propria of the rectum at least four hours post infection (pi). Replication appears to be initiated mostly in lymphoid aggregates. SIV disseminates away from the mucosa in less than four hours after rectal infection. The first target cells of SIV include T cells, but virus is also found associated with DC-SIGN^+^ cells. Presence at both sites (lymphoid aggregates and lamina propria) suggests entry via digestive epithelium as well as via follicle-associated epithelium. Prevention strategies will therefore have to cover these two sites.

## Results

### Experimental design

Rectal infections were performed with one hundred rectal Animal Infectious Dose 50 (rAID_50_). This corresponds to 7.78 log copies of viral RNA, for a final concentration of 7.31 log copies/ml of viral RNA. This is commensurate with the highest viral loads reported in human semen of 7 to 8.5 log copies/ml of viral RNA [63]–[70]. Macaques were necropsied four hours (R-H4.1 and R-H4.2), sixteen hours (R-H16.1, R-H16.2 and R-H16.3), twenty-four hours (R-H24.1 and R-H24.2), two, three and four days (R-D2.1, R-D2.2, R-D3.1, and R-D4.1) post infection and viral distribution was analyzed by nested PCR, nested RT-PCR, immunohistochemistry and co-culture with indicator cells. Plasma viral load was below detection for R-H16.1, R-H24.1, R-H24.2, R-D2.1, R-D2.2, R-D3.1 and R-D4.1. It was not measured for R-H4.1, R-H4.2, R-H16.2 and R-H16.3 as it was assumed to be below detection considering the early time point at which the macaques were necropsied.

The macaque rectum is 7 cm long, 6 cm circumference [71]. Most often the entire rectum was prepared for morphology (with alternate segments embedded in paraffin and frozen for cryosectionning). For some macaques alternate segments (representing one half of the rectum) were chemically and enzymatically disrupted to obtain cell suspensions enriched for epithelial cells (R-H4.1, R-H24.1, R-D2.2, R-D4.1) or for lamina propria cells (R-H16.3).

Peripheral blood mononuclear cells (PBMC) were purified by density centrifugation.

Individual colon-draining mesenteric lymph nodes were either processed for morphology (some embedded in paraffin and others frozen for cryosectionning) or were mechanically disrupted to obtain a suspension of mononuclear cells. Axillary lymph nodes were usually separated in two, which were processed for morphology or mechanically disrupted to yield mononuclear cells.

Three macaques were sacrificed five (R-D5.1), seven (R-D7.1) and nine (R-D9.1) days pi. This allowed us to determine that the 100 rAID_50_ dose leads to viral dissemination pattern similar to those previously observed with ten rAID_50_ (Text S1, Figure S1).

Controls included macaques infected with heat-inactivated virus and sacrificed four (M-H4.1), sixteen (M-H16.1) and twenty-four (M-H24.1) hours post inoculation, as well as two healthy rhesus macaques. Control rhesus macaques gave negative results in all assays for SIV.

### SIV enters intraepithelial cells but is not amplified in rectal epithelia

**Figure 1:**
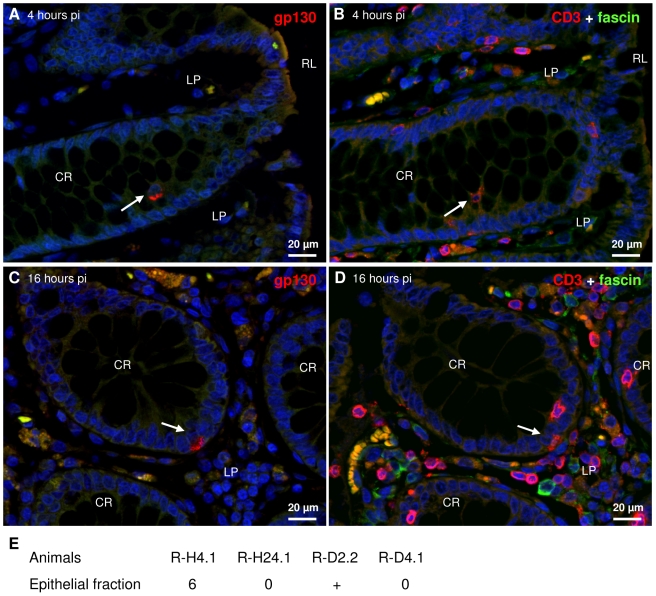
SIV is present in the rectal epithelium as early as four hours post infection. Serial paraffin sections of R-H4.1 (sacrificed four hours pi; A and B) and R-H16.2 (sacrificed sixteen hours pi; C and D) were labeled by IHF for gp130 (Alexa-546, A and C), CD3 (TRITC, B and D) or fascin (Alexa-488, B and D) and nuclei were stained with DAPI (A–D). CR, crypt; LP, lamina propria; RL, rectal lumen. Arrows on serial sections point to intraepithelial T lymphocytes positive for SIV antigens. Cell-associated virus in the epithelial cell fraction was assayed by co-culture of cells isolated from the rectum. Results are expressed as TCID_50_ per million cells. The viral load does not increase over the first four days of infection; + the TCID_50_ could not be calculated due to small number of wells positive for SIV antigen (E).

Rare SIV antigen positive (SIV^+^) intraepithelial cells were observed by immunohistofluorescence (IHF) four, sixteen and twenty-four hours pi (Figure 1, Text S1, Table S1 and Figure S2A). Some of these cells were confirmed to be T cells (Figures 1A–D). In contrast, IHF did not show SIV^+^ epithelial cells at any time point. The presence of SIV^+^ intraepithelial cells indicates that the virus could have crossed the digestive epithelium in less than four hours.

We never found SIV^+^ cells by IHF in the follicle-associated epithelium. We also never found viral DNA amplified by PCR in laser microdissected follicle-associated epithelium (microdissection described in Text S1 and Figures S3A and S3B).

**Figure 2:**
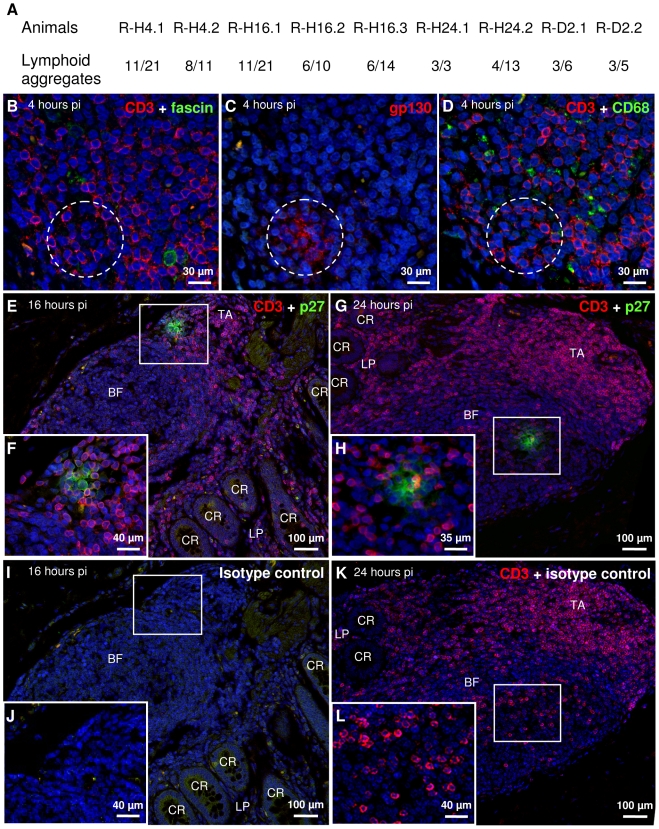
SIV is present in lymphoid aggregates of the colo-rectal mucosa starting from four hours post infection. SIV DNA is amplified by nested PCR for *gag* in most lymphoid aggregates microdissected from paraffin sections of macaques sacrificed four hours to two days pi (A); ratios indicate the number of lymphoid aggregates positive for SIV DNA relative to the total number of lymphoid aggregates tested for each macaque. Clusters of SIV-antigen positive cells are observed in paraffin embedded sections of the colo-rectal mucosa of R-H4.1 (sacrificed four hours pi; B, C and D, serial sections, dashed circle indicates the same area in the three micrographs), R-H16.1 (sacrificed sixteen hours pi; E, F enlargement of area boxed in E), and R-H24.1 (sacrificed twenty-four hours pi; G, H enlargement of area boxed in G). These clusters of SIV^+^ cells are not observed in serial sections of R-H16.1 (I, J enlargement of area boxed in I) or R-H24.1 (K, L enlargement of area boxed in K) labeled with an irrelevant antibody of the same isotype. These clusters contain T cells but not CD68^+^ macrophages or fascin^+^ DCs. Sections were labeled by IHF for gp130 (Alexa-546, C), p27 (Alexa-488, E, F, G and H), CD3 (TRITC, B, D–H, K and L), fascin (Alexa-488, B), CD68 (Alexa-488, D), irrelevant antibody (Alexa-488 I–L) and nuclei were stained with DAPI (B–L). CR, crypt; LP, lamina propria; TA, T cell area of mucosal lymphoid aggregate; BF, B cell follicle.

The epithelial cell fraction contains cells from the digestive epithelium, with less than 0.1‰ of cells coming from the follicle-associated epithelium [72]. Very little, if any, cell-associated virus was found in cells isolated from the rectal epithelium (Figure 2E), indicating that there was no viral amplification in cells of the epithelium. Cell-associated virus could correspond to infected intraepithelial T cells.

### SIV reaches mucosal lymphoid aggregates as early as four hours post infection

Four hours pi and onwards, we detected viral DNA in microdissected lymphoid aggregates in all macaques (Figure 2A, example of microdissected area in Figures S3C and S3D). Over 60% of lymphoid aggregates were positive for SIV DNA at four and sixteen hours pi (Figure 2A). Moreover, at least one third of these positive aggregates contained more than one copy of SIV DNA (with up to 20/20 PCR positive for SIV DNA, Table S2). The number of copies of SIV DNA in microdissected samples was below the detection limit of real-time PCR, preventing more accurate quantification. The majority of lymphoid aggregates remained positive for SIV at later time points (Figure 2A). In addition, lymphoid aggregates could contain SIV DNA in several serial sections suggesting local foci of infection (Table S2). The SIV DNA load of lymphoid aggregates appeared stable from four to forty-eight hours pi (Text S1, Figure S4A).

The presence of virus in lymphoid aggregates was confirmed by IHF detection of capsid (p27) and envelope (gp130) proteins. Rare clusters of adjoining cells showed labeling for SIV proteins in infected macaques four hours to two days pi (Figures 2C and 2E–H, Table S1, Figure S2B), the latest time point examined. The labeling was cytoplasmic, and appeared more punctate four hours pi (Figure 2C, dashed circle) than at later time points (Figures 2E–H). A control antibody of the same isotype as the antibodies to p27 and gp130 failed to bind on serial sections, demonstrating that binding is specific (Figures 2I–L). The number of SIV^+^ cell clusters in the lymphoid aggregates appeared to increase slightly at days one and two pi (Text S1, Table S1 and Figure S2B).

SIV^+^ cells were phenotyped. Both CD3^+^ and CD3^−^ cells were found positive for SIV antigens in the clusters (Figures 2B, 2D, 2F and 2H). SIV^+^ CD3^−^ cells were negative for fascin, a marker for mature DCs (Figure 2B) and for a macrophage-specific epitope of CD68 (Figure 2D). Early targets of SIV include therefore T cells as well as unidentified cells which do not appear to be mature DCs or macrophages.

The presence in lymphoid aggregates of viral DNA and cells positive for SIV antigens as early as four hours pi is a strong indication that entry can occur through follicle-associated epithelium. The slight increase over time suggests local viral replication.

### SIV also reaches the lamina propria four hours post infection

**Figure 3:**
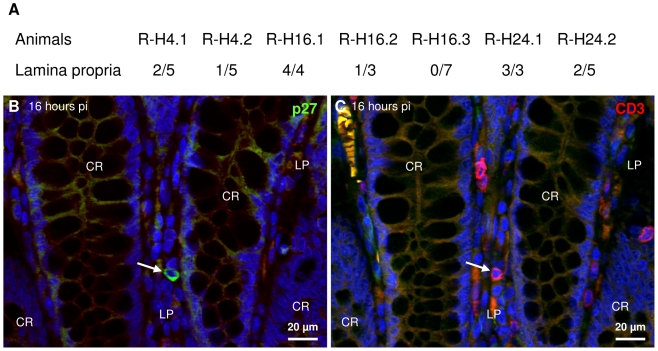
SIV is present in the colo-rectal lamina propria as early as four hours post infection. SIV DNA is amplified by nested PCR for *gag* in some lamina propria samples microdissected from paraffin sections four to twenty four hours pi (A); ratios indicate the number of lamina propria sites positive for SIV DNA relative to the total number of lamina propria sites tested for each macaque. Serial paraffin sections of R-H16.3 (sacrificed sixteen hours pi; B and C) were labeled for p27 (Alexa-488, B) and CD3 (TRITC, C) and nuclei were stained with DAPI (B and C). CR, crypt; LP, lamina propria. Arrows on serial sections point to a lamina propria T lymphocyte positive for SIV antigen.

The lamina propria was infected as early as four hours pi as shown by detection of viral DNA (Figures 3A and S4B) in laser-microdissected samples (Figures S3E and S3F). Lamina propria samples were positive for SIV in 40% of individual microdissected sample in the first day pi (Figure 3A). In contrast with lymphoid aggregates, the ratio of positive PCRs never exceeded 3/10 in the lamina propria (data not shown).

Infection of lamina propria was confirmed by IHF. Individual SIV^+^ cells were occasionally detected four hours to two days pi the latest time point examined (Figures 3B and 3C). Of note, about half of these cells were confirmed to be T cells (Figures 3B and 3C). The others were not T cells (CD3^−^), DCs (fascin^−^) or macrophages (negative for a macrophage-specific epitope of CD68) (Figures 3B and 3C and data not shown). The number of SIV antigen positive lamina propria cells appeared to decrease over time (Figure S2C).

The presence of virus in the lamina propria could indicate that the virus crossed the basement membrane in addition to the digestive epithelium in less than four hours.

### SIV mRNA production proceeds at a low level

We assayed for the presence of SIV *Env* singly spliced mRNA in tissues of all macaques sacrificed between four hours and three days pi (except R-H24.2). As RT-PCR could not be performed on microdissected samples, the entire mucosa was used for detection of spliced mRNA. It is therefore not possible in this assay to discriminate between lymphoid aggregates and lamina propria. Singly spliced mRNA was evidenced by nested RT-PCR in the rectal mucosa of R-H4.1, R-H16.2 and R-D3.1 (Table 1). It was never detected in control macaques. Detection of SIV *Env* singly spliced mRNA in the tissues of infected macaques shows that transcription and splicing proceed during the first days of rectal SIV infection.

**Table 1:** *Env* singly spliced mRNA is detected earlier in the colo-rectal mucosa than in lymph nodes.

Macaque	Colorectal mucosa	Colic lymph nodes	Axillary lymph nodes
R-H4.1	+	−	−
R-H4.2	−	−	−
R-H16.1	−	−	−
R-H16.2	+	−	−
R-H16.3	−	−	−
R-H24.1	−	−	−
R-D2.1	−	−	−
R-D2.2	−	−	−
R-D3.1	+	−	−

The singly spliced mRNA for the *Env* gene was detected by nested RT-PCR in the indicated tissues.

### Heat-inactivated virus does not lead to detection of SIV in the mucosa

Free viral DNA contained in viral stocks [73] could lead to false positive PCR results at early time points. In order to ascertain that the viral DNA detected in the mucosa of SIV inoculated macaques was due to infection, we inoculated three macaques with a quantity of heat-inactivated virus corresponding to 100 rAID_50_. We necropsied them four, sixteen and twenty-four hours after inoculation (macaques M-H4.1, M-H16.1 and M-H24.1 respectively), and analyzed the rectal mucosa. No SIV DNA was ever found in those samples despite performing over 220 individual PCR for M-H4.1, 250 for M-H16.1 and 280 for M-H24.1 at various dilutions to rule out PCR inhibition in the samples. This indicates that free viral DNA cannot enter the rectal mucosa. Therefore, the viral DNA observed four hours pi in the infected macaques is indeed due to ongoing viral replication in the rectal mucosa.

IHF for SIV antigens performed on macaques inoculated with heat-inactivated virus did not show SIV^+^ cells in the rectum of these macaques (four to six different segments were extensively sampled for each macaque). This indicates that the SIV^+^ cells observed in infected macaques correspond to live virions internalized by cells or to infected cells.

We also did not find evidence for the presence of spliced mRNA by nested RT-PCR in the macaques inoculated with heat-inactivated virus, indicating that the detection of singly spliced viral mRNA in infected macaques requires exposure to live virus.

### Mucosal DC-SIGN^+^ cells interact in vivo with SIV

**Figure 4:**
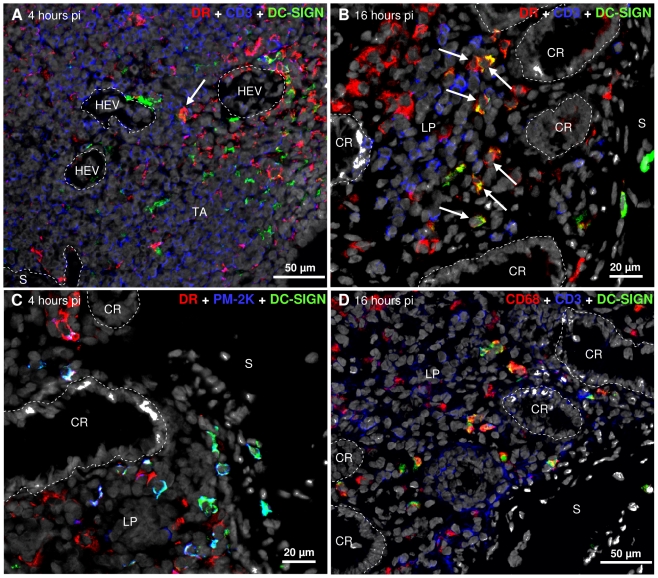
DC-SIGN^+^ cells are macrophages in the colo-rectal mucosa during the first sixteen hours of infection. DC-SIGN^+^ cells are observed in lymphoid aggregates of R-H4.2 (sacrificed four hours pi; A) and lamina propria of R-H4.1 (sacrificed four hours pi; C) and R-H16.3 (sacrificed sixteen hours pi; B and D). DC-SIGN^+^ cells express only occasionally MHC-II molecules (A and B, arrows), but co-express PM-2K (C) and CD68 (D) marking them as macrophages. Frozen sections of colo-rectal mucosa were labeled for DC-SIGN (Alexa-488), CD3 (Alexa-350, A, B and D), MHC-II Mamula-DR (Alexa-546, A, B and C) or PM-2K (Alexa-350, C), CD68 (Alexa-546, D) and nuclei were stained with TO-PRO®-3 (A–D). The computer-generated merged images are shown. CR, crypt; LP, lamina propria; S, submucosal connective tissue; TA, T cell area of mucosal lymphoid aggregate; HEV, high endothelial venule.

As DC-SIGN can bind SIV, we localized and phenotyped DC-SIGN-expressing cells by IHF early in infection. DC-SIGN^+^ cells were observed in lymphoid aggregates (Figure 4A) and in the lamina propria (Figures 4B–D), but we never could detect DC-SIGN^+^ intraepithelial cells. Almost all DC-SIGN^+^ cells co-expressed a macrophage-specific epitope of CD68, indicating that they are macrophages (Figure 4D). Most DC-SIGN^+^ cells also co-express PM-2K, marking them as mature macrophages (Figure 4C). A few DC-SIGN^+^ cells co-expressing MHC class II could carry out antigen presentation (Figures 4A and 4B).

**Figure 5:**
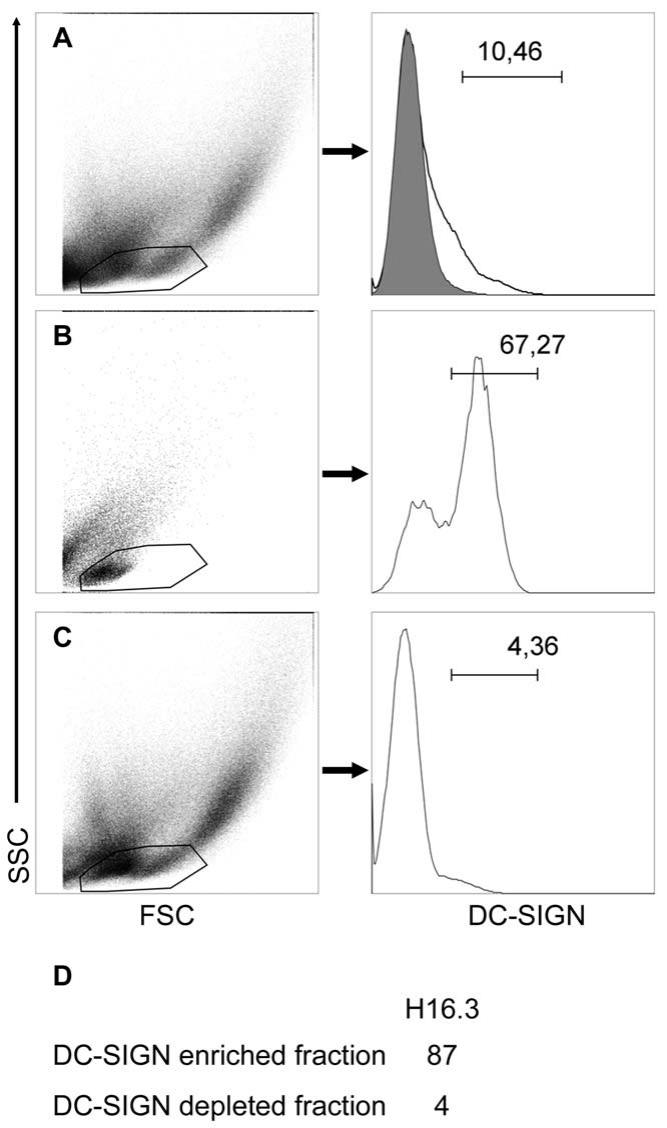
Rectal DC-SIGN^+^ cells can bind SIV sixteen hours post infection. Flow cytometry analysis of cells from the rectal collagenase fraction of R-H16.3 (sacrificed sixteen hours pi) shows enrichment of DC-SIGN^+^ cells from total cells (A) in the DC-SIGN^+^ fraction (B) and depletion in the DC-SIGN^−^ fraction (C); left dot plots show side scatter (SSC) versus forward scatter (FSC) and gate in which expression of DC-SIGN is analyzed; right histograms show expression of DC-SIGN and percentage of DC-SIGN^+^ cells (bar on histogram); gray filled histogram in A corresponds to isotype control. Cell-associated viral load (expressed as TCID_50_ per million cells; D) shows that over twenty-fold more cell-associated virus is found in the DC-SIGN enriched cell fraction than in the DC-SIGN depleted cell fraction (D).

We tested interaction of SIV with DC-SIGN^+^ cells on the rectal collagenase fraction of macaque R-H16.3 (Figure 5). The enriched DC-SIGN^+^ cell fraction (Figure 5B) carried a 20-fold higher viral load (Figure 5D) than the DC-SIGN^−^ cells (Figures 5C and 5D) from the same macaque. Nevertheless, we never observed by IHF cells positive for both SIV and DC-SIGN.

### SIV rapidly disseminates from the mucosa to the lymph nodes

Viral DNA was detected as early as four hours pi in colic lymph nodes but not prior to two days pi in axillary lymph nodes (Table 2). Cell-associated virus was found in colic lymph nodes in more than half of the macaques, and as early as four hours pi (Table 3). In contrast it was only transiently detected twenty-four hours pi in axillary lymph nodes (Table 3). Viral transcription was below detection in colic and axillary lymph nodes (Table 1).

**Table 2:** Viral DNA is detected in colic lymph nodes prior to peripheral blood and axillary lymph nodes.

Macaque	Colic lymph nodes	Axillary lymph nodes	PBMC
R-H4.1	−	−	−
R-H4.2	+	−	−
R-H16.1	−	−	−
R-H16.2	+	−	−
R-H16.3	+	−	−
R-H24.1	+	−	+
R-H24.2	−	−	+
R-D2.1	+*	+*	+
R-D2.2	+	−	+
R-D3.1	+	+*	−
R-D4.1	+	+^§^	+

The presence of viral DNA was assayed on cell suspensions, on OCT-frozen samples (*) or on paraffin sections (^§^). +: viral DNA detected; −: no viral DNA detected. PBMC: peripheral blood mononuclear cells.

**Table 3:** Cell-associated virus is detected rapidly in lymph nodes and peripheral blood.

Macaque	Colic lymph nodes	Axillary lymph nodes	PBMC
R-H4.1	2	−	−
R-H4.2	1	−	1
R-H16.1	4	−	2
R-H16.2	*	−	−
R-H16.3	−	−	*
R-H24.1	−	*	−
R-H24.2	13	*	−
R-D2.1	−	−	−
R-D2.2	9	−	*
R-D3.1	−	−	1
R-D4.1	−	−	−

Results are expressed as TCID_50_ per million cells.

*at least one well was positive for SIV antigen, but the TCID_50_ could not be calculated due to small number of SIV antigen positive wells. − no SIV antigen positive well in the experiment. PBMC: peripheral blood mononuclear cells.

Viral DNA was however detected as early as twenty-four hours pi in PBMC (Table 2). Cell-associated virus was also found in peripheral blood as early as four hours pi, albeit at a lower level (Table 3).

These data suggest that SIV disseminates in draining lymph nodes at four hours pi and to axillary lymph nodes at later time points.

## Discussion

Despite the importance of rectal infection in the AIDS epidemic, current knowledge regarding HIV rectal entry is limited to in vitro work, which does not reproduce fully the complexity and specificity of the rectal milieu. An understanding of HIV mucosal entry would be of considerable help in the design of microbicides and vaccines. We addressed the question of rectal entry in the rhesus macaque infected by SIVmac251, the animal model closest to the human infection by HIV [53], [54]. In order to increase our chances to detect virus during early acute infection, we chose a viral dose ten times higher than in our previous work [55], [56]. This dose is in the low range of the doses used for studies of vaginal transmission [74]–[78]. It is commensurate with the highest viral loads reported in the semen of HIV^+^ men [63]–[70]. This one hundred rAID_50_ dose does not affect the dissemination profile.

SIV entry is massive. If we consider our sampling to be representative of the entire rectum, one can estimate the viral DNA in the rectum of each macaque four hours pi to be on the order of 55,000 copies in lymphoid aggregates and on the order of 290,000 in the lamina propria (see Text S1 for details). As we could not find SIV DNA in mock-infected macaques, this suggests that approximately 3×10^5^ viral particles were able to cross the rectal epithelia and undergo reverse transcription, representing close to 1% of input viral particles. Some of these copies must be integrated as spliced mRNA could be found in the rectum as early as four hours pi. The much lower number of cells positive for SIV antigens (on the order of 10^3^–10^4^ per macaque, Figure S2) suggests that most copies of SIV DNA correspond to defective particles undergoing abortive infection. Cells positive for SIV antigens can be productively infected or have merely internalized viral antigens. As, in vivo, the average life cycle of SIV is under 10 hours [79], it is likely that the cells positive for SIV antigens observed sixteen hours pi and onwards are infected cells in which translation proceeds. The fraction of these cells which will go on to produce virus is currently not possible to estimate.

SIV entry is a very rapid process, as infection is established in the lamina propria, in lymphoid aggregates and in more distal sites at least at four hours pi, the earliest time point assessed. Viral entry after vaginal inoculation was shown, by in situ acidic inactivation, to be complete in less than 30 minutes [77], with reverse transcription completed as early as two hours pi [78]. The reverse transcriptase inhibitor tenofovir applied rectally two hours after rectal infection protected only one third of inoculated macaques [80], indicating that reverse transcription can be completed in less than two hours. This is faster than expected from in vitro work. However, one should note that gut lymphocytes are inflammatory but hyporesponsive [81], and that this may accelerate the viral cycle. It was not possible to narrow down further the time necessary for virus to be imported from the inoculum as, in our hands, acidic inactivation was toxic by the rectal route.

SIV entry could involve trans-epithelial transport of SIV. Early reports of HIV in rectal epithelial cells of HIV^+^ patients [82]–[84] have not been confirmed. Many now accept that infected epithelial cells are not found in vivo [44], [85]. In our previous work we had not observed infected rectocytes [56]. In the present work we could never observe infected epithelial cells either by IHF or by ISH. We did find virus at distal sites as early as four hours pi. Trans-epithelial transport is a likely explanation for this rapid dissemination, as viral production in situ is unlikely over such a short period of time. Indeed, the estimated mean intracellular phase of the life cycle for HIV in vivo is 14.4 to 21.6 hours [86]. It is shorter for SIV, with a mean life cycle of 9.4 hours [79]. The trans-epithelial transport of SIV does not appear to involve capture by DC processes extending into the rectal lumen through tight junctions. Indeed, we never observed intraepithelial DCs at early time points of infection using classical DC markers (DC-SIGN, fascin). The presence of SIV in both lymphoid aggregates and digestive mucosa four hours pi argues in favor of transcytosis of virions by both the digestive epithelium and the follicle-associated epithelium. One should note that entry appears more efficient across the follicle-associated epithelium. However the surface of the digestive epithelium is much larger than that of the follicle-associated epithelium, and the amount of virus entering through this route could be greater.

SIV structural proteins are found intracellularly by IHF in intraepithelial cells (including T cells), in lamina propria cells (T cells and non-T cells), and in T cell-containing clusters in lymphoid aggregates. The non-T cells are neither macrophages nor mature DCs as they do not express CD68, DC-SIGN or fascin. The SIV antigen positive clusters probably correspond to the infection of a single cell by virus from the inoculum. Secondary diffusion of SIV antigen to neighboring cells could occur by infection (a possibility for macaques infected sixteen hours and onwards) or by cell-cell fusion. In favor of the former is the fact that lymphoid aggregates often contain several copies of SIV DNA (Table S2). SIV^+^ cells are scarce and the detection of *Env* singly spliced mRNA is rare. These observations suggest that SIV replication is initiated in very few cells of the rectal mucosa at early stages of infection. We have no evidence for viral production in the digestive part of the mucosa. In contrast, the presence of SIV^+^ cell clusters suggests that viral production occurs in lymphoid aggregates.

During the first day of infection at least, rectal DC-SIGN^+^ cells have an overall distribution pattern similar to healthy human and macaque rectum [52], [87]. They appear less abundant in the lamina propria, but more abundant in lymphoid aggregates than Jameson et al. observed in healthy rhesus macaque mucosa [87]. They all express macrophage markers, as was described in healthy human rectum [52]. In contrast to healthy mucosa [52], [87], in early SIV infection rectal DC-SIGN^+^ cells do not all express MHC class II. DC-SIGN^+^ cells bind infectious virions, as infectious virus is enriched in the DC-SIGN^+^ cell fraction. The number of virions bound by each cell is presumably small as these cells are not positive by IHF.

SIV dissemination is rapid as cell-associated virus could be recovered from draining lymph nodes and from the peripheral blood as early as four hours pi. This indicates that virus-carrying cells have left the mucosa to percolate through the colic lymph node chain and reach the circulation in less than four hours. The very early presence of viral DNA in draining lymph nodes strongly suggests that infected cells are one means of viral dissemination from the mucosa. Virus could also travel as cell-bound virions. This is one explanation of the discrepancy observed between co-culture results and nested PCR results, the other one being a stochastic effect at very low viral loads. Finally, one cannot exclude transport as free virions in the lymph draining the rectum. To determine whether this occurs, one would have to cannulate lymph from the colon under prolonged anesthesia. This not only raises technical and ethical concerns, but could also modify exchanges between lymphoid tissues.

Rectal infection appears to involve rapid entry and reverse transcription, as has been noted for the vaginal [77], [78] and oral routes [88] but there are clear differences with these entry pathways. Dissemination after rectal entry is possibly more rapid than after oral entry, where SIV DNA is found in many lymphoid tissues twenty-four hours pi [88]. It is also more rapid than following vaginal entry, where SIV DNA could be found at very low-levels in draining lymph nodes eighteen hours pi and in some lymphoid tissues twenty-four hours pi [77]. We found that a high proportion of virus crosses the epithelium upon rectal inoculation. This is similar to oral infection [88], but contrasts with vaginal entry where only a very small proportion of the total inoculum enters the vaginal mucosa [78]. We show that T cells, but not fascin positive DCs or macrophages, are among the first targets of SIV during rectal transmission. This is not the case during vaginal transmission where fascin positive DCs are the first cells associated with SIV [77] or during oral infection where macrophages are infected [88].

The rapid kinetics of rectal entry and dissemination has important consequences for the development of preventive strategies. Rhesus macaques can be protected from rectal infection by tenofovir applied locally prior to inoculation [80]. Protection is not observed if the plasma concentration of tenofovir is below 75 ng/ml. This is in contrast with what is observed for vaginal infection, where protection is observed with plasma concentrations of tenofovir as low as 11 ng/ml [89]. This suggests that protection from rectal infection (and possibly not from vaginal infection) requires inhibition of reverse transcription in distal sites such as lymph nodes and not exclusively in the rectum. One cannot exclude that routine use of reverse transcriptase inhibitors with such plasma concentrations will lead to side effects and present risks of selection for resistant reverse transcriptase mutants. Therefore, there is a use for microbicide preparations for rectal use aiming to prevent virion interaction with the epithelium (digestive as well as follicle-associated) and entry into cells. This would complement (or replace) preparations aiming to block reverse transcription.

In conclusion, we show that SIV crosses in less than four hours both the digestive epithelium and the follicle-associated epithelium. We propose that entry occurs by transcytosis at both sites, with rectocytes and M cells being the most likely candidates to carry out transcytosis of virions. Following entry, SIV infects T cells as well as non-T cells in the mucosa. SIV initiates replication locally in the rectal lymphoid aggregates, and to a lower extent in the lamina propria. Virus is rapidly transported to distal sites as infected cells, as virions associated with cells possibly expressing DC-SIGN or as free virions present in the lymph. Reverse transcription occurs in the rectal mucosa during the first hours of infection. Reverse transcription may also occur in draining lymph nodes. To be effective against rectal transmission of HIV, a vaccine will have to induce immunity at the rectal surface, but also in distal lymphoid sites.

## Methods

### Macaques and tissue collection

Macaques were housed at the L3 animal facility of the Pasteur institute (France) in accordance with the European Community guidelines (Journal Officiel des Communautés Européennes, L358, December 18, 1986). C. Butor was granted for this protocol the authorization to experiment on live non-human primates number 006322 by the Ministère de l'Agriculture et de la Pêche in 1994, then the authorization to experiment on live non-human primates number 78-76 by the Préfecture des Yvelines in 2005. A. Couëdel-Courteille was granted the authorization to experiment on live non-human primates number 007304 by the Ministère de l'Agriculture et de la Pêche in 1997, then the authorization to experiment on live non-human primates number 75-1068 in 2005. Several steps were taken to improve animal welfare according to the recommendations of the Weatherall report. Animals were housed in individual cages to prevent viral transmission, but up to twenty animals were housed in a single room allowing sight and sound contact with each other. Diet was supplemented with a variety of fresh fruit. All manipulations of animals were performed under ketamine anesthesia, according to regulations in France. Macaques were sacrificed by a lethal dose of pentothal under ketamine anesthesia. In addition, the tissues obtained after necropsies are currently used for another study in order to reduce the total number of animals used. Nineteen adult male rhesus macaques (*Macaca mulatta*) were used in this study. Fourteen macaques were inoculated atraumatically by the rectal route with 100 rAID_50_ [90] and otherwise as previously described [55], [56]. This corresponds to 18,000 TCID_50_ and 7.78 log copies of viral RNA [91] for a final concentration of 7.31 log copies/ml of viral RNA. The SIVmac251 viral stock originally obtained from R Desrosiers was a kind gift of A-M Aubertin. Macaques were sacrificed four hours (animals R-H4.1 and R-H4.2), sixteen hours (R-H16.1, R-H16.2 and R-H16.3), twenty-four hours (R-H24.1, R-H24.2), two days (R-D2.1, R-D2.2) and three, four, five, seven and nine days (R-D3.1, R-D4.1, R-D5.1, R-D7.1 and R-D9.1) pi. Three animals were inoculated with an identical volume of the viral stock after heat inactivation (56°C for 30 min) and euthanized four, sixteen or twenty-four hours post inoculation (M-H4.1, M-H16.1 and M-H24.1 respectively). Two healthy rhesus macaques were used as controls. Peripheral blood was collected on heparin or EDTA prior to euthanasia. Lymph nodes, colon and rectum were collected separately under sterile conditions and processed as previously described [56], omitting the Percoll gradient on the epithelial and collagenase fractions.

### Titration of cell-associated virus

Titrations were performed as previously described [55], [56]. For macaques R-H4.1 R-H4.2, R-H16.2 and R-H16.3 we used the commercial kit 〈〈Innotest® HIV Antigen P24〉〉 (Innogenetics, Gent, Belgium) which cross-reacts with the SIV p27 capsid protein instead of our homemade ELISA. The TCID_50_ was calculated according to Reed and Muench [92].

### *In situ* hybridization

ISH was performed as previously described [56] using either INT-BCIP or NBT-BCIP as substrate for alkaline phosphatase.

### Immunohistofluorescence

Five µm-thick formaldehyde-fixed paraffin-embedded sections collected on glass slides were deparaffinized and rehydrated. Antigen retrieval was performed by pressure cooking the sections for 10 min in 0.01 M buffered sodium citrate solution (pH 6). Sections were then rinsed with calcium free Dulbecco's phosphate buffered saline (PBS). Seven µm-thick cryosections were fixed for 10 min in cold acetone and rinsed in PBS.

Both types of sections were incubated for 30 min with blocking buffer (2% normal goat serum and 5% bovine serum albumin in PBS), incubated for 60 min with primary antibodies, washed in PBS 0.5% Tween20 (Sigma, St. Louis, MO), incubated with secondary antibodies for 30 min, washed, counterstained with 4,6-diamidino-2-phenylindol (DAPI) (Molecular Probes, Cergy Pontoise, France, paraffin sections) or TO-PRO®-3 iodide (Invitrogen, Cergy Pontoise, France, cryosections) and mounted in Fluoromount-G (Southern Biotechnology, Birmingham, AL).

Primary antibodies were polyclonal rabbit anti-CD3 (DAKO, Trappes, France) mouse monoclonal antibodies to fascin (clone 55K-2, IgG1, DAKO), SIV gp130 envelope protein (clone KK46, IgG1, obtained from the NIH), SIV p27 capsid protein (IgG1, Advanced Biotechnologies Inc Columbia, MD), *Aspergillus niger* glucose oxidase (isotype control, IgG1, clone DAK-GO1, DAKO), HLA-DR (clone TÜ36, IgG2b, BD Biosciences), DC-SIGN (clone 120612, IgG2a, R&D Systems, Lille, France), DC-SIGN (clone 120507, IgG2b, R&D Systems), CD68 (clone KP1, IgG1, DAKO, Trappes, France), and tissue macrophage (clone PM-2K, IgG1, AbD Serotec, Düsseldorf, Germany). Secondary antibodies were TRITC-conjugated goat anti-rabbit antibody (Southern Biotechnology) and isotype-specific (IgG1, IgG2a and IgG2b) goat anti-mouse secondary antibodies (Molecular Probes) conjugated to Alexa 350, Alexa 488 or Alexa 546.

### Image capture and analysis

IHF sections were examined under an inverted epifluorescence microscope Axiovert 200 M (Zeiss), equipped with a HBO flexible fluorescence lamp, a black and white CCD camera (Roper Scientific Coolsnap HQ) and coupled to video imaging using the Axiovision 4.4 software (Zeiss). ISH sections were examined under a DMRB (Leica) with a DC300F camera (digital module R, Leica) and the IM1000 software (Leica). Images were digitally acquired with a 20× or a 40× objective, then we used both the Axiovision software and the Photoshop software (Adobe Systems Incorporated) to analyze the different stainings.

### Laser capture microdissection of rectal mucosa

Microdissection was performed on an automated system for diode ultraviolet laser cutting and infrared laser capture of tissue samples, mounted on a Nikon Eclipse TE2000 inverted microscope equipped with a color CCD camera and coupled to video imaging. Formaldehyde-fixed paraffin-embedded 5-µm-thick sections were mounted on a polyethylene foil slide (SL Microtest GmbH, Jena, Germany) and counterstained with hematoxyline. Sections were observed on a screen using a 10× or a 20× objective. An incision path was drawn on the screen and multiple overlapping laser pulses dissected the selected tissue area. The target tissue was removed from the slide with isolation caps. The efficiency of the laser capture microdissection was assessed by examining the tissue harvested under the microscope. DNA was extracted by adapting the DNeasy blood and tissue kit protocol from QIAGEN (Courtaboeuf, France) for small DNA amounts.

### Detection of SIV viral DNA

DNA was extracted from 5×10^6^ cells in suspension using the DNeasy blood and tissue kit (QIAGEN) and from paraffin sections using the DNeasy tissue kit (QIAGEN) according to the manufacturer's instructions. To assess the quality of the extracted DNA (presence of cellular DNA, lack of PCR inhibitor), we performed a PCR for actin using sense (5′ GGG TCA GAA GGA TTC CTA TG 3′) and antisense (5′ GGT CTC AAA CAT GAT CTG GG 3′) actin primers (Genset, Paris, France). To assess the presence of viral DNA, we used either a semi-nested PCR protocol as previously described [55], [93] or a single PCR protocol with primers F-GAG-Ni-5′-CCG TCA GGA TCA GAT ATT GCA and R-GAG-Ci-5′-TTC GTA CCC AGC CCC TTC AGC in 2.5 mM MgCl_2_. Ten to twenty individual PCRs were performed on mucosa samples. Negative PBMC and lymph node samples were tested at varying dilutions to rule out potential inhibition of the PCR due to suboptimal ratios between the DNA and the primers in the sample. The detection threshold was one to two copies as assessed with serial dilution of gag containing plasmids in different volumes of DNA extracts obtained from PBMC or from microdissected tissues of uninfected macaques.

### Purification of DC-SIGN-expressing cells from the rectum

DC-SIGN-expressing cells were enriched using anti-phycoerythrin (PE) magnetic beads (Miltenyi Biotec) according to manufacturer's recommendations. Briefly lamina propria rectal cells were stained with an anti-DC-SIGN-PE antibody (clone 120507, R&D Systems), washed in MACS buffer (Miltenyi Biotec), incubated with anti-PE beads, then separated in a LS column (Miltenyi Biotec). The column was washed with MACS buffer and DC-SIGN-expressing cells were eluted. Both positive and negative fractions were washed, analyzed by flow cytometry and used to quantify cell-associated virus. Flow cytometry was performed on a FACSCalibur or a FACSCanto (BD Biosciences) and analyzed with FlowJo 8.8.6 software (TreeStar).

### Detection of *Env* singly spliced mRNA

Messenger RNA was extracted with the MicroPoly(A) Purist kit (AMBION, Courtaboeuf, France) according to manufacturer's instructions from samples of colorectal mucosa, colic and axillary lymph nodes (1 mm^3^) frozen in OCT. To assess the presence of *Env* singly spliced mRNA, we used a nested RT-PCR protocol. After a reverse transcription step, cDNAs were first PCR amplified with primers LTR-SIV-SD-5′-CGA CGG AGT GCT CCT ATA AA (located before the splice donor in the long terminal repeat region in 5′) and V1V2-Out3-5′-GAA GAG ACC ACC ACC TTA GAA (located before the Rev response element). A second PCR was performed on the initial PCR products with primers V1V2-In5-5′-AGG ATG TAT GGC AAC TCT TTG A and V1V2-In3-5′-CAC AAG ACT CTT GGA TAA CAG AA. All PCRs were performed in 3.5 mM MgCl_2_ with 10 minutes initial denaturation at 95°C, then 35 cycles of 30 seconds at 94°C, 30 seconds at 60°C, and 5 minutes at 72°C.

## Supporting Information

Figure S1 **SIV dissemination after high dose rectal infection reaches colon draining lymph nodes prior to axillary lymph nodes.** Cell-associated virus in tissues expressed as TCID_50_ per million cells shows more than one log difference between draining lymph nodes and other lymphoid tissues; + the TCID_50_ could not be calculated due to small number of wells positive for SIV antigen (A). SIV DNA amplified by nested PCR for *gag* is always found in draining lymph nodes, but not in other lymphoid tissues of R-D9.1: + viral DNA amplified, − no viral DNA amplified (B). Infected cells are detected by *in situ* hybridization for SIV in colic lymph node of R-D5.1 (C), rectal mucosa of R-D7.1 (D) and central mesenteric lymph node of R-D9.1 (E). C, INT-BCIP substrate, no counterstain; D and E NBT-BCIP substrate, eosin counterstain. Arrows point to infected cells in lymph nodes (C and E) and in the T cell area of mucosal lymphoid aggregates (D) and arrowheads to infected cells in the lamina propria (D). CR, crypt; LP, lamina propria; S, submucosal connective tissue; TA, T cell area of mucosal lymphoid aggregate; GC, germinal center; FM, follicular mantle; BF, B cell follicle; PC, parafollicular cortex.
(TIF)

Figure S2 **SIV-antigen positive elements during the first two days of infection.** SIV-antigen positive intraepithelial cells (A), clusters in lymphoid aggregates (B) or lamina propria cells (C) were counted on sections labeled by IHF. Total values were computed per macaque.
(TIF)

Figure S3 **Laser capture microdissection allows separate sampling of follicle-associated epithelium, lymphoid aggregates and lamina propria.** Figure shows paraffin sections counterstained with hematoxylin and micrographed with an Eclipse TE2000 inverted microscope before (A, C, E) and after (B, D, F) laser capture microdissection. The red dashed line corresponds to the laser pattern. The area microdissected in A and B was follicle associated epithelium (FAE), in C and D lymphoid aggregate (LA) and in E and F lamina propria (LP).
(TIF)

Figure S4 **Number of copies of SIV DNA during the first two days of infection.** The presence of SIV DNA was measured by semi-quantitative PCR on microdissected lymphoid aggregates (A) and lamina propria (B). See text for details of calculations.
(TIF)

Table S1 **Number of SIV antigen positive cells or cell clusters in colo-rectal segments during the first two days post infection.**
(DOC)

Table S2 **PCR analysis of SIV DNA on serial sections of colo-rectal lymphoid aggregates.**
(DOC)

Text S1 (DOC)
