# Supplementary figures and images for: Rapid Dissemination of SIV Follows Multisite Entry after Rectal Inoculation
Source: PLoS One. 2011 May 9;6(5):e19493. doi: 10.1371/journal.pone.0019493 (PMC3090405; doi:10.1371/journal.pone.0019493)

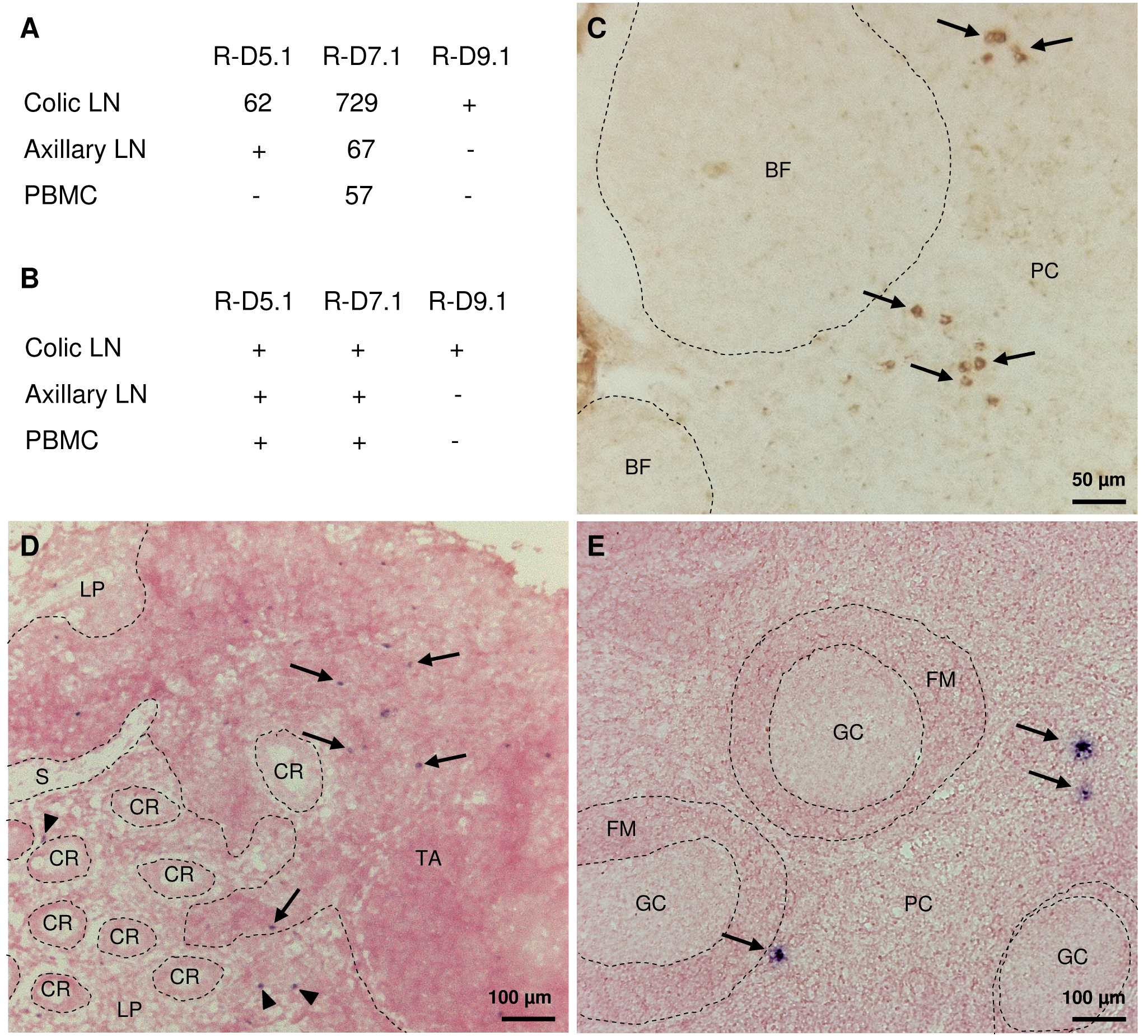

Supplement: Figure S1 — SIV dissemination after high dose rectal infection reaches colon draining lymph nodes prior to axillary lymph nodes. Cell-associated virus in tissues expressed as TCID50 per million cells shows more than one log difference between draining lymph nodes and other lymphoid tissues; + the TCID50 could not be calculated due to small number of wells positive for SIV antigen (A). SIV DNA amplified by nested PCR for gag is always found in draining lymph nodes, but not in other lymphoid tissues of R-D9.1: + viral DNA amplified, − no viral DNA amplified (B). Infected cells are detected by in situ hybridization for SIV in colic lymph node of R-D5.1 (C), rectal mucosa of R-D7.1 (D) and central mesenteric lymph node of R-D9.1 (E). C, INT-BCIP substrate, no counterstain; D and E NBT-BCIP substrate, eosin counterstain. Arrows point to infected cells in lymph nodes (C and E) and in the T cell area of mucosal lymphoid aggregates (D) and arrowheads to infected cells in the lamina propria (D). CR, crypt; LP, lamina propria; S, submucosal connective tissue; TA, T cell area of mucosal lymphoid aggregate; GC, germinal center; FM, follicular mantle; BF, B cell follicle; PC, parafollicular cortex. (TIF) [file pone.0019493.s001.tif]

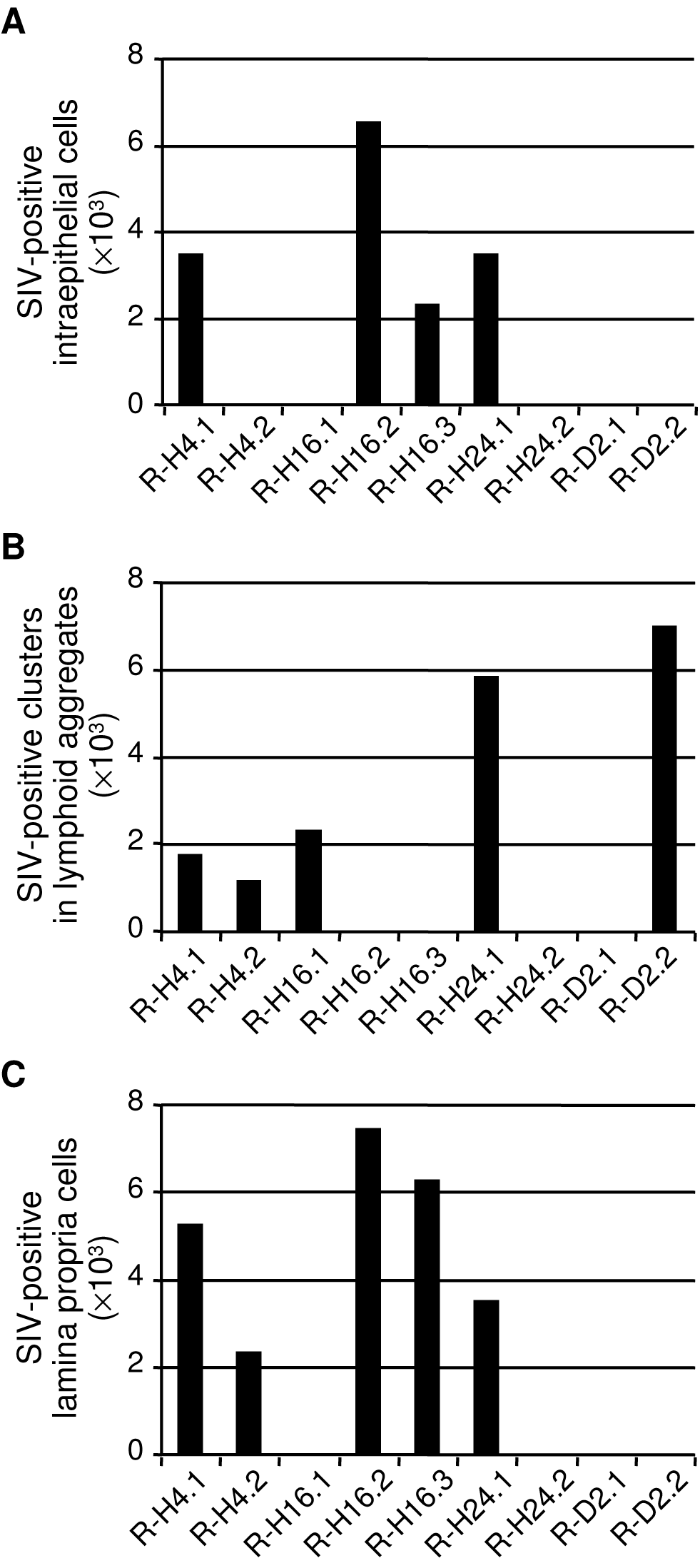

Supplement: Figure S2 — SIV-antigen positive elements during the first two days of infection. SIV-antigen positive intraepithelial cells (A), clusters in lymphoid aggregates (B) or lamina propria cells (C) were counted on sections labeled by IHF. Total values were computed per macaque. (TIF) [file pone.0019493.s002.tif]

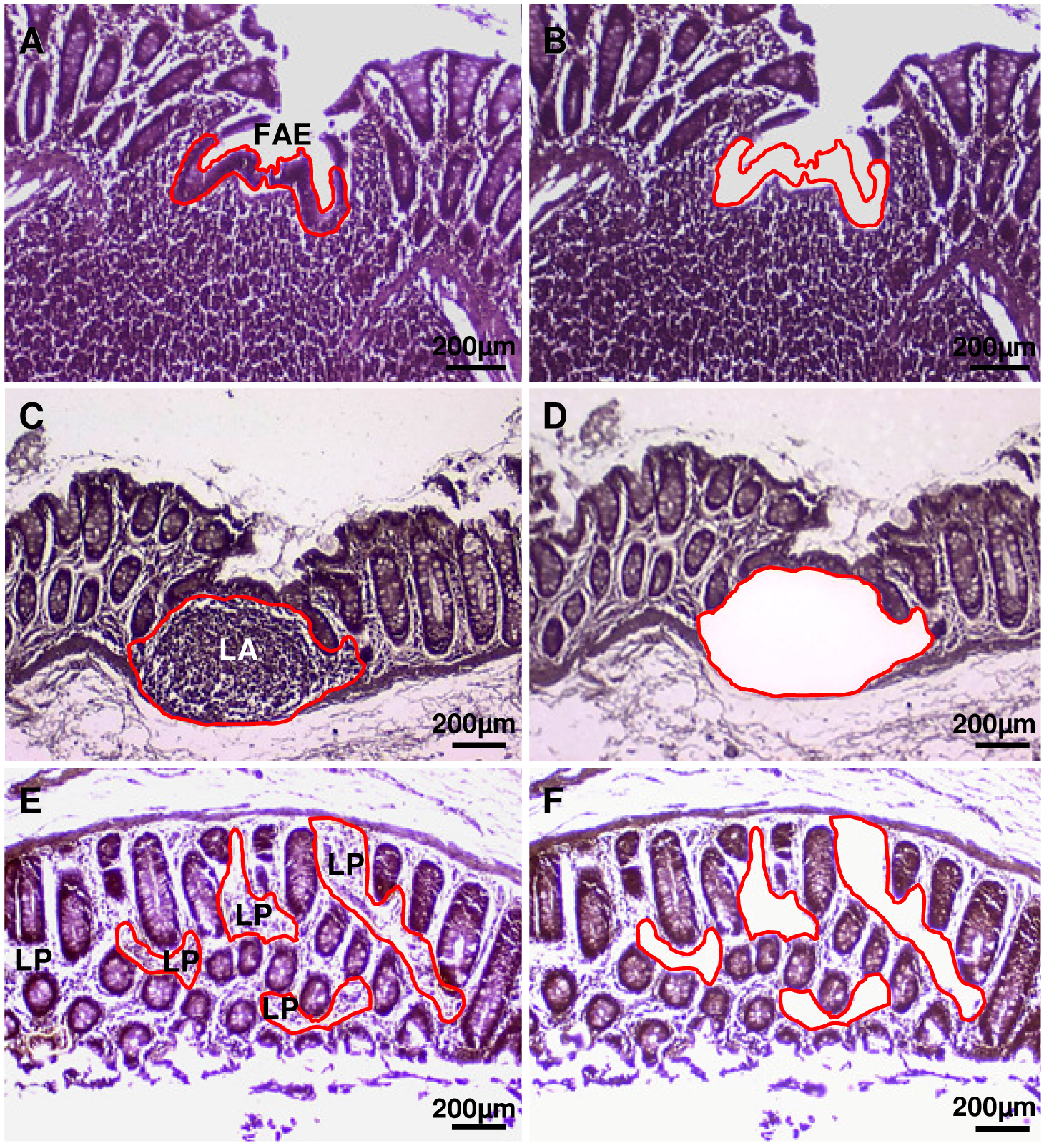

Supplement: Figure S3 — Laser capture microdissection allows separate sampling of follicle-associated epithelium, lymphoid aggregates and lamina propria. Figure shows paraffin sections counterstained with hematoxylin and micrographed with an Eclipse TE2000 inverted microscope before (A, C, E) and after (B, D, F) laser capture microdissection. The red dashed line corresponds to the laser pattern. The area microdissected in A and B was follicle associated epithelium (FAE), in C and D lymphoid aggregate (LA) and in E and F lamina propria (LP). (TIF) [file pone.0019493.s003.tif]

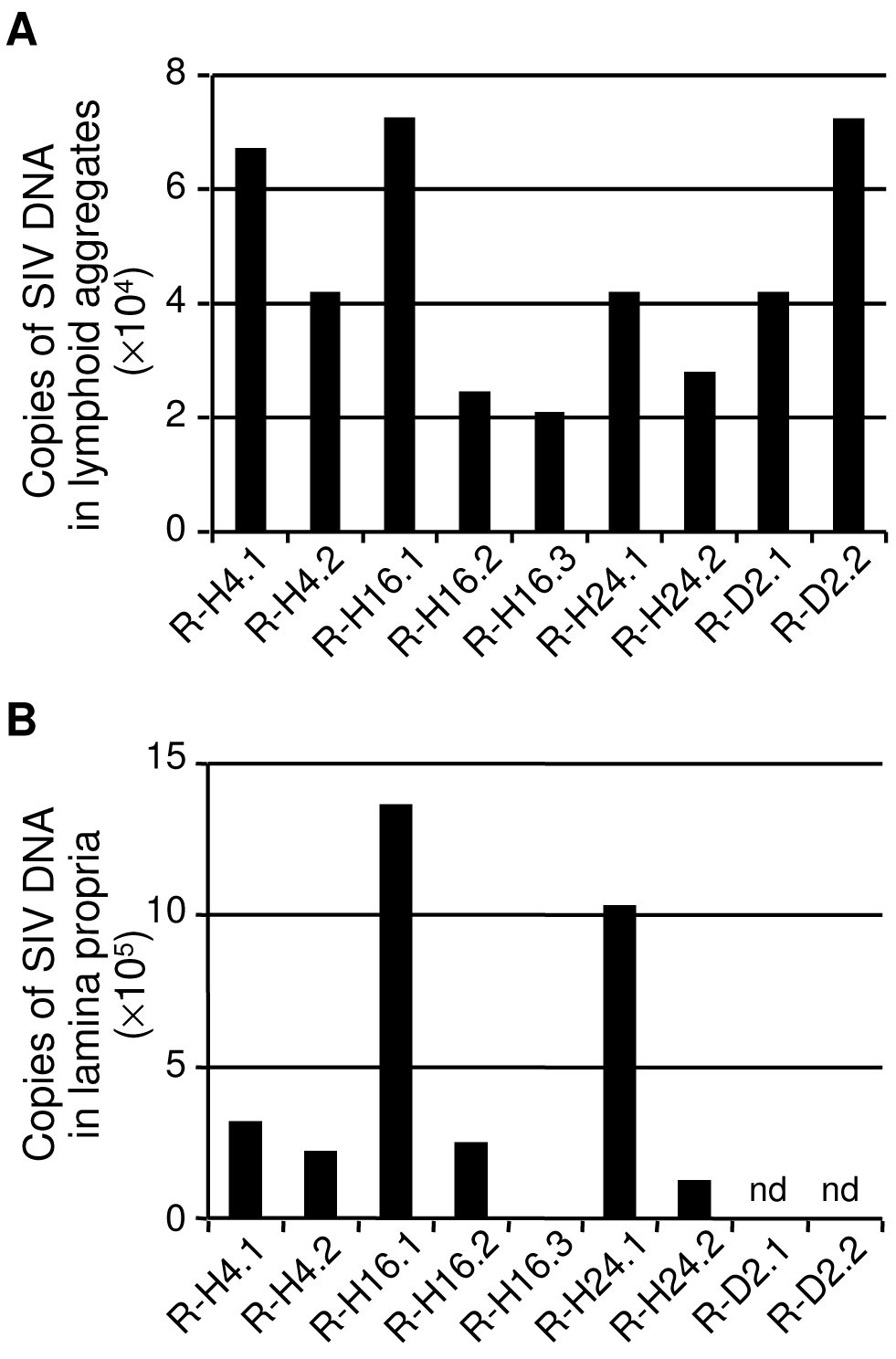

Supplement: Figure S4 — Number of copies of SIV DNA during the first two days of infection. The presence of SIV DNA was measured by semi-quantitative PCR on microdissected lymphoid aggregates (A) and lamina propria (B). See text for details of calculations. (TIF) [file pone.0019493.s004.tif]
