# Supplementary material for: Rapid Dissemination of SIV Follows Multisite Entry after Rectal Inoculation
Source: PLoS One. 2011 May 9;6(5):e19493. doi: 10.1371/journal.pone.0019493 (PMC3090405; doi:10.1371/journal.pone.0019493)
Supplement: Table S1 — Number of SIV antigen positive cells or cell clusters in colo-rectal segments during the first two days post infection. (DOC) [file pone.0019493.s005.doc]

**Table S1. Number of SIV antigen positive cells or cell clusters in colo-rectal segments during the first two days post infection.**

|  |  |  | Average number per segment of | | |
| --- | --- | --- | --- | --- | --- |
| Macaque | Segment | Number of sections | intraepithelial cells | clusters in lymphoid aggregates | lamina propria cells |
| R-H4.1 | 1 | 2 | 1 | 0.5 | 1.5 |
|  | 2 | 1 | 0 | 0 | 0 |
|  | 3 | 1 | 0 | 0 | 0 |
|  | 4 | 2 | 0 | 0 | 0 |
| R-H4.2 | 1 | 2 | 0 | 0 | 0 |
|  | 2 | 2 | 0 | 0.5 | 0 |
|  | 3 | 1 | 0 | 0 | 0 |
|  | 4 | 1 | 0 | 0 | 0 |
|  | 5 | 1 | 0 | 0 | 1 |
|  | 6 | 1 | 0 | 0 | 0 |
| R-H16.1 | 1 | 1 | 0 | 0 | 0 |
|  | 2 | 2 | 0 | 0.5 | 0 |
|  | 3 | 2 | 0 | 0 | 0 |
| R-H16.2 | 1 | 1 | 0 | 0 | 0 |
|  | 2 | 3 | 2.33 | 0 | 2.67 |
|  | 3 | 4 | 0 | 0 | 0 |
|  | 4 | 1 | 0 | 0 | 0 |
|  | 5 | 1 | 0 | 0 | 0 |
| R-H16.3 | 1 | 5 | 1 | 0 | 2.2 |
|  | 2 | 3 | 0 | 0 | 0 |
|  | 3 | 3 | 0 | 0 | 0 |
|  | 4 | 1 | 0 | 0 | 0 |
|  | 5 | 2 | 0 | 0 | 0.5 |
|  | 6 | 2 | 0 | 0 | 0 |
| R-H24.1 | 1 | 4 | 0.75 | 0.75 | 0.25 |
|  | 2 | 2 | 0 | 0.5 | 0.5 |
|  | 3 | 1 | 0 | 0 | 0 |
| R-H24.2 | 1 | 1 | 0 | 0 | 0 |
|  | 2 | 2 | 0 | 0 | 0 |
|  | 3 | 1 | 0 | 0 | 0 |
|  | 4 | 2 | 0 | 0 | 0 |
| R-D2.1 | 1 | 1 | 0 | 0 | 0 |
| R-D2.2 | 1 | 1 | 0 | 0 | 0 |
|  | 2 | 1 | 0 | 1 | 0 |
