# Supplementary material for: Rapid Dissemination of SIV Follows Multisite Entry after Rectal Inoculation
Source: PLoS One. 2011 May 9;6(5):e19493. doi: 10.1371/journal.pone.0019493 (PMC3090405; doi:10.1371/journal.pone.0019493)
Supplement: Table S2 — PCR analysis of SIV DNA on serial sections of colo-rectal lymphoid aggregates. (DOC) [file pone.0019493.s006.doc]

**Table S2. PCR analysis of SIV DNA on serial sections of colo-rectal lymphoid aggregates.**

|  | aggregate 1 | | aggregate 2 | | aggregate 3 | | aggregate 4 | |
| --- | --- | --- | --- | --- | --- | --- | --- | --- |
| Macaque | PCR* | Surface area^§^ | PCR* | Surface area^§^ | PCR* | Surface area^§^ | PCR* | Surface area^§^ |
| R-H4.1 | 3/19 | 1,570,227 |  |  |  |  |  |  |
|  | 1/16 | 165,319 |  |  |  |  |  |  |
| R-H16.1 | 18/19 | 911,936 | 0/19 | 356,385 | 5/20 | 271,479 | 3/18 | 325,672 |
|  | 2/5 | 963,062 | 0/6 | 366,895 | 2/19 | 257,824 | 1/11 | 272,640 |
|  | 5/16 | 1,018,823 | 0/3 | 263,250 |  |  |  |  |
|  | 3/20 | 1,073,837 | 0/19 | 234,762 |  |  |  |  |
|  | 3/18 | 1,081,338 |  |  |  |  |  |  |
| R-H16.2 | 4/19 | 1,830,843 |  |  |  |  |  |  |
|  | 1/18 | 584,712 |  |  |  |  |  |  |
| R-D2.2 | 0/19 | 1,165,556 |  |  |  |  |  |  |
|  | 2/18 | 969,406 |  |  |  |  |  |  |
|  | 3/8 | 905,645 |  |  |  |  |  |  |
|  | 20/20 | 873,506 |  |  |  |  |  |  |

* results are expressed as number of PCRs positive for SIV antigens over total number of PCRs performed for the sample. ^§^ expressed in µm^2^.
