# Supplementary material for: Rapid Dissemination of SIV Follows Multisite Entry after Rectal Inoculation
Source: PLoS One. 2011 May 9;6(5):e19493. doi: 10.1371/journal.pone.0019493 (PMC3090405; doi:10.1371/journal.pone.0019493)
Supplement: Text S1 — (DOC) [file pone.0019493.s007.doc]

**Text S1**

**Infection with a higher viral dose does not change dissemination patterns**

We had used in our previous work ten rectal Animal Infectious Dose 50 (rAID_50_) for our viral dissemination studies [55,56]. In the present study we wanted to increase our chances to localize virus early in infection. Rectal infections were therefore performed with one hundred rAID_50_. Even if this dose is commensurate with the highest viral loads reported in human semen [63-70], we wanted to determine whether this higher inoculum would modify the viral dissemination pattern. We therefore sacrificed three animals (R-D5.1, R-D7.1 and R-D9.1) five, seven and nine days post-infection (pi). This is similar to our previous work, where animals were sacrificed 7, 10, 11 and 14 days pi.

As with the ten rAID_50_ dose, wide variations were observed between animals (Figure S1). Animals R-D5.1 and R-D7.1 had at least one log more cell-associated virus in colic lymph nodes than in axillary lymph nodes (Figure S1A). All tissues assayed for these two animals were positive for viral DNA (Figure S1B). Infected cells were observed by *in situ* hybridization (ISH) in colic but not in axillary lymph nodes. They were found in the cortical area for R-D5.1 (Figure S1C), and throughout the lymph nodes for R-D7.1 (data not shown). Infected cells were also detected in the rectal mucosa (lamina propria and lymphoid aggregates) in R-D7.1 (Figure S1D). Animal R-D9.1 had a very low level of infection. Nevertheless, we observed cell-associated virus (Figure S1A) as well as viral DNA (Figure S1B) in colic lymph nodes and infected cells in central mesenteric lymph nodes (Figure S1E).

The viral dissemination pattern is similar for high and low viral doses: initial confinement of viral replication to draining lymph nodes, then colonization of the mucosa and finally of axillary lymph nodes [55,56]. According to the classification previously proposed [55], animals R-D5.1 and R-D9.1 were at stage 2 of viral dissemination (draining lymph nodes but not distal lymph nodes are positive for SIV by ISH with infected cells restricted to the cortex) and animal R-D7.1 was at stage 3 (draining lymph nodes but not distal lymph nodes are positive for SIV by ISH with infected cells throughout the draining lymph nodes).

We were therefore comforted to use the high viral dose (100 rAID_50_) for all new infections.

**The total number of SIV antigen positive elements appears to increase in lymphoid aggregates and to decrease in other parts of the mucosa over the first two days of infection**

The macaque rectum is 7 cm long, 6 cm circumference [71]. It contains therefore approximately 14,000 sections of 5 µm thickness in its length. We included in the analysis some segments from the lower colon, as they would be exposed to the inoculum (there is no valve between the colon and the rectum). To compensate we did not include segments very close to the anus. We refer to the area examined as rectum for the sake of simplicity in the calculations of total numbers of infected cells par macaque. The segments of mucosa that we harvested for morphology are random. If we assume that these segments are representative, we can extrapolate from our data the total number of SIV-antigen positive cells per rectum. The estimate of 14,000 sections per rectum may be an overestimate, as some shrinking occurs during paraffin embedding. However, relative values will be correct. When we had several sections for a given segment, we averaged the values obtained for each segment to have a representative value for the segment. As the distribution is bimodal (there is virus or there is no virus), an estimate of the standard error of the mean is of no interest, and is not included.

The average number of SIV-antigen positive cells or clusters per segment is given in Table S1. We then calculated an estimate per macaque (Figure S2).

**Laser capture microdissection allows precise excision of microanatomical areas in macaque mucosa**

We chose laser capture microdissection to sample micro-anatomically defined regions of the colo-rectal mucosa. The incision path was drawn on the screen coupled to the microscope. This allowed very precise definition of the region to be microdissected (Figure S3A, C and E). After harvesting of the excised area, the section was examined again to confirm proper excision (Figure S3B, D and F). The surface of the area microdissected was automatically computed by the system.

**The total number of copies of SIV DNA appears stable over the first two days of infection**

The total number of copies of SIV DNA per rectum was computed using the same principles as for the total number of SIV^+^ cells. In the paragraphs below, numbers above 10,000 have rounded to the next hundred. The number of copies present in an area harvested by microdissection is computed as one copy per positive PCR. The values presented here are therefore underestimates, as in some cases a positive PCR reaction will contain several copies of SIV DNA.

We analyzed all aggregates larger than 50,000 µm^2^ in a given segment. The total surface analyzed was 59,438,300 µm^2^ or lymphoid aggregates in 104 segments, of which 19,500,600 µm^2^ were four hours pi. Total numbers of copies of SIV DNA were computed per macaque (Figure S4A). For some aggregates, we analyzed serial sections (Table S2). Lymphoid aggregates could be positive for SIV DNA over several sections, and in one case negative over several sections, suggesting foci of infection.

For the lamina propria, it was not technically possible to harvest the entire lamina propria on a given section. We therefore measured by image analysis the surface area occupied by the lamina propria on sections (excluding sections tangential to the rectum). Our estimate is 40,971,700 µm^2^ of lamina propria per rectal circumference. To obtain an estimate of the number of copies for a given animal, we divided the total number of positive PCRs by the total surface of the microdissected areas, multiplied by 40,971,700 and by 14,000 (Figure S4B).
